# The Therapeutic Effect and Mechanism of *Lactobacillus gardneri* on Nonalcoholic Fatty Liver Disease

**DOI:** 10.4014/jmb.2501.01051

**Published:** 2025-12-29

**Authors:** Keyan Zhu, Jie Xiang, Fengqin Lu, Jiabo Wang, Yu Zhang, Haibo Feng, Xiaoxing Xiang

**Affiliations:** 1 Clinical Medical College of Yangzhou University, Yangzhou, Jiangsu, 225001, P.R. China; 2 Department of Pharmacology, School of Medicine, Yangzhou University, Yangzhou, Jiangsu, 225009, P.R. China; 3 Jiangsu Key Laboratory of Integrated Traditional Chinese and Western Medicine for Prevention and Treatment of Senile Diseases, Yangzhou University, Yangzhou, Jiangsu, 225001, P.R. China; 4 Reproductive center, The First People’s Hospital of Huai’an, Huai’an, Jiangsu, 223001, P.R. China; 5 Orthopedics department, Huai’an 82 Hospital, Huai’an, Jiangsu, 223001, P.R. China; 6 Department of Gastroenterology, Northern Jiangsu People's Hospital, Clinical Medical College of Yangzhou University, Yangzhou, Jiangsu, 225009, P.R. China

**Keywords:** Non-alcoholic fatty liver disease, *Lactobacillus gasseri* SBT2055, lipid accumulation, intestinal homeostasis

## Abstract

This study aimed to investigate the effect of *Lactobacillus gasseri* SBT2055 (LG2055) on non-alcoholic fatty liver disease (NAFLD). Mice were fed with high-fat diet (HFD) to establish NAFLD animal model. HFD mice were administrated with LG2055 gavage to explore the role of LG2055 on NAFLD. This study revealed that LG2055 gavage decreased serum levels of alanine aminotransferase (ALT), aspartate aminotransferase (AST), total cholesterol (TC), and triglyceride (TG), reduced lipid accumulation in liver tissue, promoted the expression of intestinal mucosal proteins mucin 2 (MUC2), defensin alpha 1 (DEFA1), and defensin alpha 4 (DEFA4), inhibited the levels of pro-inflammatory factors tumor necrosis factor-alpha (TNF-α) and interleukin (IL)-6, and increased the levels of anti-inflammatory factors IL-10 and immunoglobulin (Ig)A, IgG, and IgM. LG2055 decreased the abundance of *Verrucomicrobota*, *Akkermansiaceae* and *Akkermansia* while increased the abundance of *Bacteroidota*, *Actinobacterota* and *Muribaculaceae*. These findings implied that LG2055 alleviate liver damage caused by NAFLD by reducing hepatic lipid accumulation and gut homeostasis and regulating gut microbiota to inhibit intestinal inflammation, and increase immune regulation.

## Introduction

Non-alcoholic fatty liver disease (NAFLD), as a chronic liver disease associated with modern lifestyle and dietary changes, has attracted extensive attention in recent years [1]. NAFLD is caused by the accumulation of excess lipids in the liver, leading to lipotoxicity that may develop into non-alcoholic steatohepatitis, liver fibrosis, and even hepatocellular carcinoma [2]. Diet modification, lifestyle interventions, weight loss, and the treatment of the underlying metabolic syndrome remain the main forms of treatment, but long-term adherence is required to be effective [3, 4]. Therefore, more effective strategies are needed to delay or mitigate NAFLD.

The gut microbiota is composed of many different microbial populations in the gastrointestinal tract of mammals, which is a key factor to maintain physiological intestinal homeostasis, resist pathogens and regulate the immune system [5]. Disruption of gut microbiota homeostasis may lead to gastrointestinal diseases, neurodegenerative diseases, cardiovascular diseases, and metabolic diseases [6, 7]. The imbalance of gut microbiota is one of the causes of NAFLD [8-10]. *Lactobacillus rhamnosus* alleviates NAFLD by increasing the concentration of short-chain fatty acids in intestinal tract and regulating energy metabolism and lipid metabolism [11]. *Bifidobacterium* intake helps to reduce pathological inflammatory response in NAFLD mice, while reducing lipid and blood sugar levels [12]. *Lactobacillus gasseri* SBT2055 (LG2055) is a probiotic lactobacillus isolated from human feces [13]. By stimulating energy expenditure, SBT2055 improved glucose tolerance and reduced weight gain in rats [14]. In addition, LG2055 induced the production of immunoglobulin in the gut of mice and reduced the inflammatory state in the serum of diabetic rats [15, 16]. However, the potential role of LG2055 in NAFLD needs to be further explored.

In this study, we constructed a NAFLD mouse model to explore the potential effect of LG2055 in the treatment of NAFLD by monitoring changes of serum markers levels, analyzing lipid metabolism, and staining liver tissue with oil red O and HE. This study aims to provide a scientific basis for the accurate treatment of NAFLD and is expected to provide useful references for clinical treatment research.

## Material and Methods

### Animals

C57BL/6J mice (male, 7-8 weeks, 20 ± 2 g) were purchased from the Charles River (China). All mice were fed adaptively for one week to adapt to the experimental environment. The experiments were carried out with the approval of the Ethics Committee of Yangzhou University.

### NAFLD Modeling and Grouping

For NAFLD modeling, mice were fed with high-fat-diet (HFD, 60% energy from fat) for 12 weeks to induce hepatic steatosis. The mice fed with normal diet (ND, only 10% energy form fat) were used as control (*n* = 4). The HFD mice were randomly divided 3 groups: HFD group (*n* = 4); HFD+PBS group (*n* = 4), HFD mice were given phosphate buffered saline (PBS) intragastric administration at 9^th^ week for 4 weeks; HFD+LG2055 group (*n* = 4), HFD mice were given 1 × 10^8^ CFU/ml *Lactobacillus paragasseri* SBT2055 (LG2055, the Milk Science Research Institute, Megmilk Snow Brand Co. Ltd., Japan) intragastric administration at 9^th^ week for 4 weeks. The LG2055 was prepared according to Nakayama Y *et al*. [17]. All mice were raised in an environment with a temperature of 23°C, humidity of 50%, and a light/dark cycle of 12 h. The preparation of LG2055 referred to Eguchi K *et al*. [18].

### Specimen Collection

Blood samples of mice were collected at different time points (including before LG2055 administration and every week during LG2055 administration) using the tail vein sampling method. The collected blood was centrifuged at 1,850 rpm/min for 5min. After centrifugation, the upper serum samples were carefully transferred to a new centrifuge tube and stored at -80°C. After 12 weeks, mice were euthanized with excessive pentobarbital sodium to collect liver and intestine tissues.

### Liver Function Detection

The levels of liver damage markers alanine aminotransferase (ALT), aspartate aminotransferase (AST), lipid metabolism markers total cholesterol (TC), triglyceride (TG), liver fibrosis marker hyaluronic acid (HA), and oxidative stress marker malondialdehyde (MDA) in serum were detected by ELISA Kit for ALT (SEA207Mu, USCN, China), ELISA Kit for AST (SEB214Mu, USCN), TC Content Assay Kit (BC1985, Solarbio, China), Amplex Red TG Assay Kit (S0219M, Beyotime, China), Mouse HA ELISA Kit (CSB-E08121m, Cusabio, China), and MDA Assay Kit (S0131M, Beyotime), respectively, in according to the manufacturer's certificate.

### Histopathology of Liver Tissues

Paraffin sections were prepared for hematoxylin-eosin (HE) staining and frozen sections were prepared for oil red O staining. For HE staining, paraffin sections were dewaxed, rehydrated, and then stained with hematoxylin for 5 min and eosin for 1 min. After dehydration and vitrifaction, the sections were sealed and observed under a microscope. For oil red O staining, frozen sections were fixed in 4% paraformaldehyde at room temperature for 30 min and then washed in 70% ethanol for 5 sec. Sections were stained in oil red stain solution for 10 min, washed with 70% ethanol, and then stained with hematoxylin for 2 min. After washing with running water, sections were sealed and observed under a microscope.

### QRT-PCR

The total RNA was extracted from intestine tissues using Trizol reagent (Thermo Fisher Scientific, USA) and digested by DNA-free kit (Ambion, USA). The reverse transcription was performed using PrimeScriptTM RT Master Mix (Takara, Japan). RT-PCR was performed using the Absolute Q-PCR SYBR Green Supermix (Bio-Rad, USA). Actb was used as the internal reference. The primer sequences were: MUC2 forward 5’-GTCCTGACC AAGAGCGAACA-3’ and reverse 5’-ACAGCACGACAGTCTTCAGG-3’; DEFA1 forward 5’-AGAAGAGGA CCAGGCCGTAT-3’ and reverse 5’-TGGTCTCCATGTTCAGCGAC-3’; DEFA4 forward 5’-AGAACGAGT TCGTGGGACTT-3’ and reverse 5’-GTCATCTGCATGTTCAGCGG-3’; Actb forward 5’-TGAGCTGCGTTT TACACCCT-3’ and reverse 5’-GCCTTCACCGTTCCAGTTTT-3’.

### Western Blot

Total protein was extracted from intestine tissues using the RIPA buffer (Beyotime) and the concentration of samples was detected by the BCA Protein Quantification Kit (E112-02, Vazyme, China). Samples were separated on 10% SDS-PAGE and transferred to the PVDF membranes (Millipore, Massachusetts, USA). After blocking by 5% nonfat milk, membranes were incubated with primary antibodies anti-MUC2 (27675-1-AP, Proteintech, China), anti-DEFA1 (PA5-117076, Thermo Fisher Scientific), anti-DEFA4 (PA5-103124, Thermo Fisher Scientific) and GAPDH (10494-1-AP, Proteintech) overnight at 4°C and incubated another 2 h with Goat Anti-Rabbit IgG H&L (HRP) (Abcam, UK) at room temperature. The bands were visualized by the BeyoECL Plus (Beyotime) and analyzed using ImageJ software (National Institutes of Health, USA).

### ELISA Detection

The levels of immune globulin IgA, IgG, IgM and inflammation factors TNF-α, IL-6, IL-10 in intestine tissues were detected by ELISA kits. The intestine tissues were washed with cold PBS to remove digestive fluid. Then, intestine tissues were homogenated with lysate for 30 sec in the ice bath and incubated for 10 min on ice. Centrifugation was carried out under 4°C for 10 min at 10,000 rpm to separate supernatant. The protein concentration was detected by BCA method. ELISA kits for detection were ELISA Kit for IgA (SEA546Mu, USCN, China), ELISA Kit for IgG (CEA544Mu, USCN), ELISA Kit for IgM (CEA543Mu, USCN), Mouse TNF-α ELISA Kit (PT512, Beyotime), Mouse IL-6 ELISA Kit (PI326, Beyotime) and Mouse IL-10 ELISA Kit (PI523, Beyotime).

### Gut microbiota Analysis by 16S rRNA

Fecal samples from each group of mice were subjected to total DNA extraction using the QIAamp PowerFecal Pro DNA Kit (Qiagen). The DNA was quantified using Nanodrop method, and the quality of DNA extraction was checked by 1.2% agarose gel electrophoresis. After amplification, PCR products were quantified by Quant-iT PicoGreen dsDNA Assay Kit (Thermo Fisher Scientific) using microplate reader (BioTek, FLx800). Equal proportions of each sample were taken and mixed together to form a template, and then the library index sequence and splicing sequence required for Illumina sequencing were added for a second PCR. After library purification, the samples were tested and precisely quantified by the High Sensitivity DNA Kit (Agilent Technologies Inc.) using bioanalyzer (Agilent Technologies Inc.). The libraries were subjected to 250 bp paired-end sequencing on a MiSeq System (Illumina) according to the standard procedure. The possible pathways associated with gut microbiota were analyzed through PICRUSt2 analysis.

### Statistic Analysis

Data were presented as mean ± SD and analyzed using GraphPad Prism 8.0 (GraphPad Software, USA). Student’s *t*-test was used for comparison between two groups and one-way ANOVA was used for comparisons among different groups. *p* < 0.05 was considered statistically significant.

## Results

### LG2055 Alleviates Hepatic Damage of HFD-Fed Mice

**Fig. 1:**
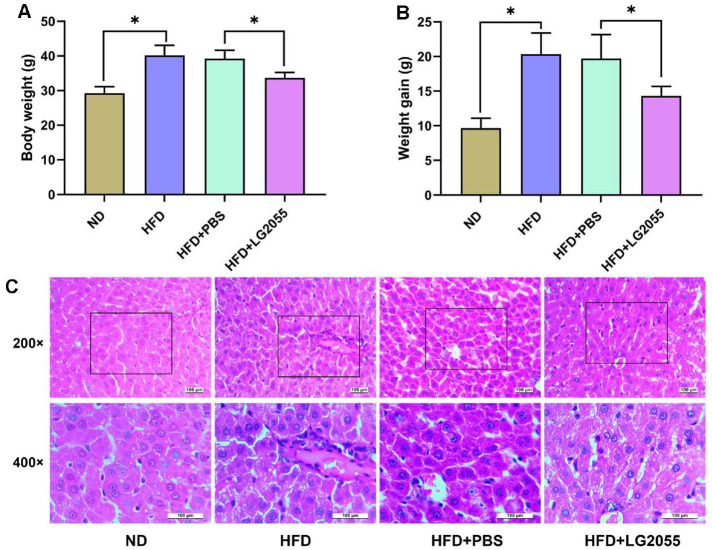
LG2055 alleviates hepatic damage of HFD-fed mice. (**A**) The weight changes of mice in each group (**B**) The weight gain of mice in each group (**C**) The pathological changes of liver tissues. *p < 0.05.

Compared with the ND group, the HFD group mice showed increased body size and weight gain. After treatment with LG2055, the weight of mice significantly decreased (Fig. 1A and 1B). HE staining detected the pathological changes of liver tissues in each group, as shown in Fig. 1C. The liver tissue structure of the ND group was intact without obvious fat vacuoles. In HFD and HFD+PBS groups, the cells are arranged irregularly, with many fat vacuoles and hyperplasia of connective tissue in the portal area. Compared with the HFD+PBS group, the HFD+LG2055 group showed a decrease in liver tissue steatosis and hyperplasia of connective tissue.

### LG2055 Improves Hepatic Function of HFD-Fed Mice

**Fig. 2:**
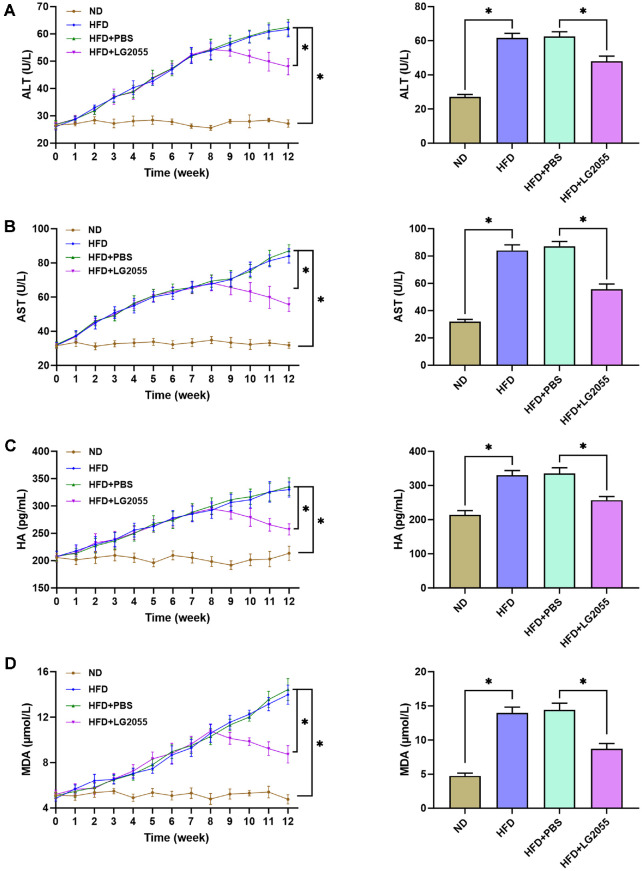
LG2055 improves the hepatic function of HFD-fed mice. (**A**) The changes of serum ALT in mice of each group (**B**) The changes of serum AST in mice of each group (**C**) The changes of serum HA in mice of each group (**D**) The changes of serum MDA in mice of each group. *p < 0.05.

To investigate the role of LG2055 on liver function of HFD-fed mice, changes of the serum levels of ALT, AST, HA, and MDA were detected after LG2055 treatment. As the duration of HFD increased, the levels of ALT, AST, HA, and MDA in HFD-fed mice gradually increased (Fig. 2A-2D), indicating that HFD caused cell damage, fibrosis, and oxidative stress to liver. After gavage with LG2055, serum levels of ALT, AST, HA, and MDA were decreased gradually, suggesting that LG2055 significantly improved the liver damage of HFD-fed mice.

### LG2055 Regulates Lipid Metabolism of Liver of HFD-Fed Mice

**Fig. 3:**
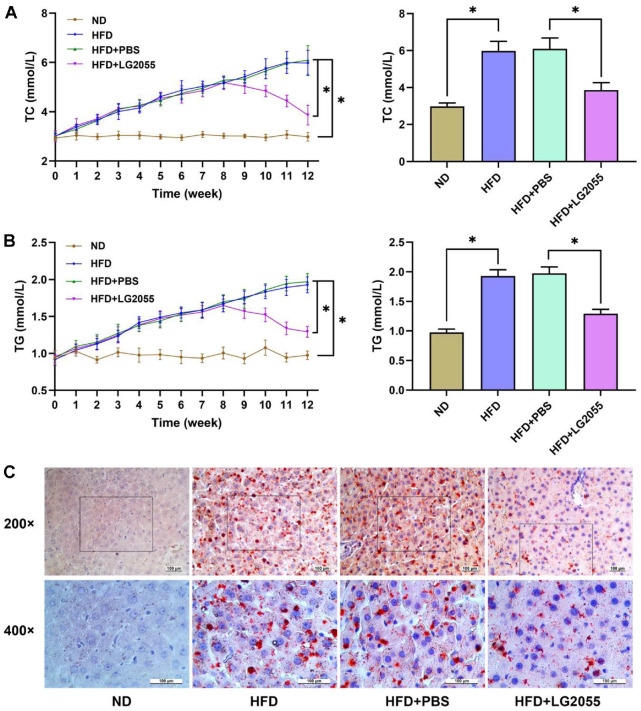
LG2055 regulates lipid metabolism of the liver of HFD-fed mice. (**A**) The serum TC in mice of each group (**B**) The serum TG in mice of each group; (**C**) The lipid accumulation in liver tissue. *p < 0.05.

HFD is an important factor affecting liver lipid metabolism. The changes of serum TC and TG were detected to investigate the effect of LG2055 on lipid metabolism. Continuous feeding of HFD resulted in a gradual increase in serum TC and TG levels. The levels of serum TC and TG were gradually reduced by gavage with LG2055 (Fig. 3A and 3B), indicating that LG2055 could reduce blood lipid levels. Lipid accumulation in liver tissue was then observed by oil red O staining (Fig. 3C). There was no fat deposition in the liver tissue of ND mice. In HFD group and HFD + PBS group, there were obvious fat depositions, uneven arrangement of hepatocytes, and vacuolar necrosis in liver tissue. The liver fat deposition was significantly reduced after LG2055 gavage.

### LG2055 Regulates Intestinal Homeostasis of HFD-Fed Mice

**Fig. 4:**
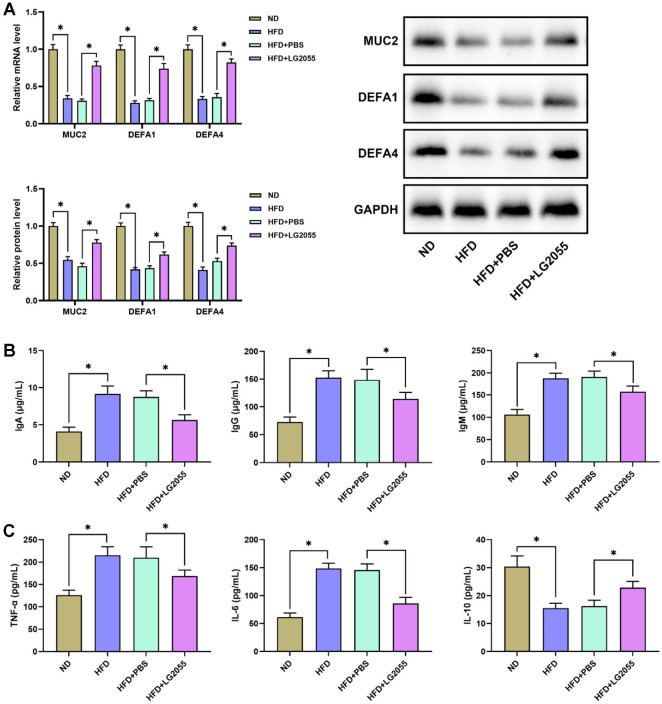
LG2055 regulates intestinal homeostasis of HFD-fed mice. (**A**) The levels of MUC2, DEFA1, and DEFA4 in intestine tissues (**B**) The levels of immune globulin IgA, IgG, IgM in intestine tissues (**C**) The levels of TNF-α, IL-6, IL-10 in intestine tissues. *p < 0.05.

The levels of MUC2, DEFA1 and DEFA4 in intestine tissues were detected by PCR and western blot. The results showed that the expression levels of MUC2, DEFA1 and DEFA4 in intestine tissues of HFD-fed mice were markedly lower than the intestine tissues of ND-fed mice. After LG2055 gavage, the expression levels of MUC2, DEFA1, and DEFA4 increased significantly (Fig. 4A). The changes of immune globulin IgA, IgG, IgM and inflammation factors TNF-α, IL-6, IL-10 in intestine tissues were then detected by ELISA kits. HFD feeding decreased levels of IgA, IgG and IgM, but increased levels of TNF-α, IL-6 and IL-10 in intestine tissues (Fig. 4B). Obviously, LG2055 gavage increased levels of IgA, IgG and IgM, while decreased levels of TNF-α, IL-6 and IL-10 in intestine tissues (Fig. 4C). These above data indicated that LG2055 enhanced intestinal homeostasis and decreased intestinal inflammation of HFD-fed mice.

### LG2055 Regulates Intestinal Flora of HFD-Fed Mice

**Fig. 5:**
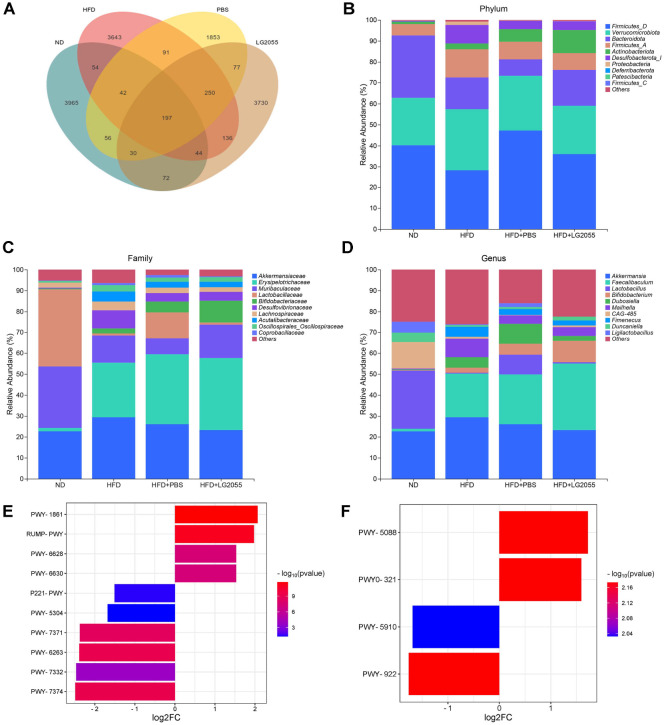
LG2055 regulates intestinal flora of HFD-fed mice. (**A**) The Venn diagram of OTU (**B**) Analysis of speciesrelated abundance at the phylum level; (**C**) Analysis of species abundance at the scientific level; (**D**) Species abundance analysis at the genus level; E. Analysis of differential metabolic pathways in intestinal flora between ND group and HFD group of mice. PWY-1861: Formaldehyde assimilation II (RuMP Cycle); RUMP-PWY: Formaldehyde oxidation I; PWY-6628: Super pathway of L-phenylalanine biosynthesis; PWY-6630: Super pathway of L-tyrosine biosynthesis; P221-PWY: Octane oxidation; PWY- 5304: Super pathway of sulfur oxidation (*Acidianus ambivalens*); PWY-7371: 1,4-dihydroxy-6-naphthoate biosynthesis II; PWY-6263: Super pathway of menaquinol-8 biosynthesis II; PWY-7332: Super pathway of UDP-N-acetylglucosamine-derived O-antigen building blocks biosynthesis; PWY-7374: 1,4-dihydroxy-6-naphthoate biosynthesis I. F. Analysis of differential metabolic pathways in intestinal flora between HFD+PBS group and HFD+LG2055 group of mice. PWY-5088: L-glutamate degradation VIII (to propanoate); PWY0-321: Phenylacetate degradation I (aerobic); PWY-5910: Mevalonate pathway I; PWY-922: Super pathway of geranylgeranyl diphosphate biosynthesis I (via mevalonate).

In order to analyze the effect of LG2055 on the gut microbiota composition of NAFLD mice, the microbiota composition and abundance were compared at the phylum, family, and genus levels and subjected to significant analysis. The Venn diagram shows the OTU overlap between groups (Fig. 5A). At the phylum level, the dominant bacterial communities are *Firmicutes*, *Verrucomicrobiota*, and *Bacteroidota*. Compared with the ND group, the HFD group and HFD+PBS group had a higher abundance of *Verrucomicrobiota* and lower abundance of *Bacteroidota* (Fig. 5B). However, compared with the HFD+PBS group, the abundance of *Bacteroidota* and *Actinobacterota* was higher (*p* < 0.05). At the family level, the dominant strains of gut microbiota are *Akkermansiaceae*, *Erysipelotrichaceae*, and *Muribaculaceae*. Compared with the ND group, the abundance of *Akkermansiaceae* and *Erysipelotrichaceae* increased in the HFD and HFD+PBS groups, while the abundance of *Muribaculaceae* decreased (Fig. 5C). However, compared with the HFD+PBS group, the abundance of *Akkermansiaceae* decreased and the abundance of *Muribaculaceae* and *Bifidobacteriaceae* increased in the HFD+LG2055 group. At the genus level, the dominant strains of gut microbiota are *Akkermansia* and *Faecalibaculum*. Compared with the ND group, the abundance of *Akkermansia* and *Faecalibaculum* increased in the HFD group and HFD+PBS group; After treatment with LG2055, the abundance of *Akkermansia* decreased, while the abundance of *Bifidobacterium* increased (Fig. 5D).

In addition, we predicted possible pathways related to gut microbiota through PICRUSt2 analysis. We selected representative metabolic pathways for the HFD group compared to the ND group and the HFD+LG2055 group compared to the HFD+PBS group based on screening criteria (adjust *p* value < 0.05, |log2 fold change| > 1.5) (Fig. 5E and 5F). Compared with the ND group, the HFD group showed an increase in the abundance of formal assimilation II (RuMP cycle), formal oxidation I, superpathway of L-phenylalanine biosynthesis, and superpathway of L-tyrosine biosynthesis, while the abundance of octane oxidation, superpathway of sulfur oxidation (*Acidanus ambivalens*), 1,4-dihydroxy-6-naphthoate biosynthesis II, superpathway of menaquinol-8 biosynthesis II, superpathway of UDP-N-acetylglucosamine derived O-antigen building blocks biosynthesis , and 1,4-dihydroxy-6-naphthoate biosynthesis I decreased (Fig. 5E). Compared with the HFD+PBS group, the HFD+LG2055 group showed an increase in the abundance of L-glutamate degradation VIII (to proparate) and phenylacetate degradation I (aerobic), while the abundance of mevalonate pathway I and superpathway of geranylgeranyldiphosphate biosynthesis I (via mevalonate) decreased (Fig. 5F).

## Discussion

NAFLD is characterized by excessive accumulation of triglycerides (TG) in the liver and is the most common liver disease [19]. The prevalence of NAFLD is increasing at an alarming rate, with prevalence rates as high as 34%in Asia [20]. This study found the anti NAFLD effect of LG2055 in mice, indicating the potential of LG2055 administration in combating the development of NAFLD.

In this study, an animal model of NAFLD was constructed by feeding HFD to explore the effects of LG2055 on the liver of NAFLD. Fat deposition in liver tissue and increased serum levels of TC and TG in HFD-fed mice indicating the success of NAFLD modeling. The results showed that LG2055 could reduce the weight and alleviate liver tissue damage caused by HFD. The monitoring results of serum ALT and AST also showed that LG2055 gavage significantly reduced the levels of ALT and AST increased by HFD feeding. The liver is the main place for the intake, storage and output of lipids with self-evident role in fat metabolism [21]. Normally, only a small amount of fatty acids are stored in liver cells as TG [22]. Excess nutrition or obesity can lead to excessive accumulation of TG in liver cells, resulting in NAFLD [21]. LG2055 is a probiotic extracted from human feces that can reduce abdominal obesity in adults [23, 24], promote fecal fat excretion [25], and lower fasting and postprandial blood lipid levels in patients with hypertriglyceridemia [26]. These studies indicate that LG2055 has therapeutic significance for diseases caused by excessive fat accumulation. In addition, NAFLD is a significant cause of liver fibrosis [27]. HA, synthesized by hepatic stellate cells, is a crucial component of the extracellular matrix in tissues [28]. In patients with NAFLD, HA serves as an indicator of fibrosis presence [29, 30]. Research findings indicate that the level of HA in HFD-fed mice was significantly elevated. However, treatment with LG2055 markedly reduced serum HA levels, suggesting that LG2055 may have a therapeutic effect on liver fibrosis. Furthermore, we observed that LG2055 also decreased the level of MDA. It has been reported that oxidative stress contributes to the onset and progression of NAFLD [31]. The inhibitory effect of LG2055 on serum MDA levels indicates that LG2055 can mitigate oxidative stress in HFD-fed mice. All these results indicate that LG2055 exhibited therapeutic effect on liver function injury and lipid metabolism of liver in NAFLD.

The gut is the largest interface between the body and the external environment. The intestinal barrier is affected by the composition of gut microbiota, diet, and intestinal inflammatory mediators [32]. HFD and bacterial infection will cause the destruction of intestinal barrier and then lead to the occurrence and progression of metabolic and autoimmune system diseases [33]. Increasing studies have found that the gut microbiota is associated with the etiology of obesity-related disorders such as NAFLD [34]. Studies have showed that LG2055 decreased bacterial colonization of *Porphyromonas gingivalis* and reduced the secretion of inflammation factors [18, 35]. MUC2 is a gel-like mucin located on the apical surface of intestinal epithelial cells, serving as a physical barrier that protects these cells from bacterial contact [36]. DEFA1 and DEFA4 are crucial antimicrobial peptides secreted by intestinal Paneth cells, which help safeguard the intestine from pathogenic bacteria [37]. In this study, modeling NAFLD resulted in a significant downregulation of MUC2, DEFA1, and DEFA4 levels. However, LG2055 gavage increased the levels of MUC2, DEFA1 and DEFA4 in intestine tissues of HFD-fed mice, indicating that LG2055 could contribute to the maintaining of intestinal barrier in NAFLD mice. Besides, LG2055 has been shown to alleviate the subjective symptoms of the common cold by improving the host immune system in immune-weakened individuals [38]. Our study Our study revealed that HFD modeling led to an increase in pro-inflammatory factors IL-6 and TNF-α in the intestine, a decrease in the anti-inflammatory factor IL-10, and an increase in the secretion of immunoglobulins IgA, IgG, and IgM. LG2055 was able to inhibit the inflammatory response in the intestine and reduce the secretion of immunoglobulins. These findings suggest that LG2055 may help repair the intestinal mucosal barrier by promoting intestinal homeostasis, thereby reducing the entry of harmful substances into the bloodstream, alleviating liver inflammation, and mitigating. NAFLD.

The gut microbiota is a complex and dynamic community composed of bacteria, archaea, eukaryotes, viruses, and parasites, comprising 500-1,000 species and 1014 bacterial species [39-41]. A healthy gut environment is regulated by the delicate balance of gut microbiota, metabolites, and host immune system. Multiple studies have shown that the pathogenesis of human NAFLD is closely related to the imbalance of gut microbiota [42, 43]. Among these microbial communities, research mainly focuses on bacteria [44]. Recent studies have found that *Lactobacillus plantarum* JM1 can balance the gut microbiota structure by increasing the abundance of beneficial bacteria and reducing the abundance of harmful bacteria [45]. The study by Li X *et al*. demonstrated that *Lactobacillus rhamnosus* RW2014 altered the gut microbiota composition of HFD rats [46]. The study by de Moura E Dias M *et al*. also showed that *Lactobacillus agalactiae* LG-G12 can increase the relative abundance of allobaculum, Bifidobacterium genus, and Prevotella in the intestine[47]. In this study, it was found that LG2055 intervention can increase the relative abundance of Bacteroidota in the gut of NAFLD mice, decrease the relative abundance of *Verrucomicrobiota*, and reduce the relative abundance of Akkermansia at the genus level. In addition, the functional prediction results showed the most significant differences in enriched abundance pathways between the ND group and the HFD group, as well as between the HFD+PBS and HFD+LG2055 groups, including formal assignment II (RumP cycle), formal oxidation I, pathway of L-phenylalanine biosynthesis, pathway of UDP-N-acetylglucosamine derived O-antigen building blocks biosynthesis, and 1,4-dihydroxy-6-naphthoate biosynthesis I, L-glutamate degradation VIII (to prop), phenylacetate degradation I (aerobic), mevalonate. TE pathway I and superpath of geranylpyrophosphate biosynthesis I (via mevalonate) pathways. From the above results, it can be concluded that LG2055 intervention may alleviate NAFLD symptoms by altering the relative abundance of gut microbiota and regulating the enrichment of related pathways. However, due to the modeling method, sample size, and short intervention time, LG2055 intervention did not completely reverse the gut microbiota dysbiosis in NAFLD.

## Conclusion

In conclusion, LG2055 reduces hepatic lipid accumulation, inhibits intestinal inflammation, and enhances immune regulation by improving hepatic lipid metabolism, restoring gut homeostasis, and modulating the gut microbiota. These actions contribute to the alleviation of liver damage caused by NAFLD. Our study provides theoretical support for the treatment of NAFLD. However, this study has some shortcomings. The mechanism of LG2055 in the treatment of NAFLD requires further exploration. Additionally, the relationship between liver function and intestinal homeostasis necessitates more in-depth investigation. The therapeutic efficacy of LG2055 in treating NAFLD still needs to be validated in clinical trials.
